# Comparative analysis of impacted upper canines: Panoramic radiograph Vs Cone Beam Computed Tomography

**DOI:** 10.4317/jced.53652

**Published:** 2017-10-01

**Authors:** Catarina-Luís-Vicente-Rodrigues Pico, Francisco-José-Fernandes do Vale, Francisco-José-Santiago-Fernandes-Amado Caramelo, Ana Corte-Real, Sónia-Margarida-Alves Pereira

**Affiliations:** 1 MS, DDS, Faculty of Medicine, University of Coimbra, Coimbra, Portugal; 2 MS, DDS, PhD, Department of Orthodontics, Dentistry Area, Faculty of Medicine, University of Coimbra, Coimbra, Portugal; 3 Laboratory of Biostatistics and Medical Informatics, Faculty of Medicine, University of Coimbra, Coimbra, Portugal; 4 MS, DDS, PhD, Department of Forensic Dentistery, Dentistry Area, Faculty of Medicine, University of Coimbra, Coimbra, Portugal

## Abstract

**Background:**

The use of CBCT exam in the study of IMC is not new. However, it’s still not known in what specific aspects CBCT exam shows a better result than then conventional exams. The aim of this study was to compare and conclude in what way the opinion regarding upper canine impaction differed when observing a panoramic image compared to the observation of a set of CBCT reconstructions.

**Material and Methods:**

Twenty patients (10 males and 10 females) with a total of 28 impacted maxillary canines were identified from the database of the Department of Dentistry, Faculty of Medicine, University of Coimbra. For each canine, two different images were available: a panoramic image and a set of CBCT reconstructions. After a random distribution of both groups images, nine orthodontists completed a questionnaire where they were asked to evaluate ten different questions regarding canine impaction. Statistic analysis was performed using Cronbach’s alpha statistics, Kappa statistics and McNemar test, considering *p*<0,05 statistically significant.

**Results:**

This study showed differences between the two images regarding tooth position. A statistical significant poor agreement was found between the two methods for the mesio-distal position of the apex (k=0,388, *p*<0,001) and for the labio-palatal tip cusp position (k=0,035, *p*=0,114). The adjacent root resorption showed a poor and very poor agreement between the two methods. Every other items were scored with an agreement between modalities ranging from moderate to strong.

**Conclusions:**

The analyses of panoramic images versus CBCT images reconstructions provided different information regarding tooth position (especially concerning the mesio-distal apex position and the labio-palatal cusp position) but also in the assessment of root resorption. Further investigation should be done to determine in what cases CBCT exam has a clear advantage over conventional 2D exams, justifying its use.

** Key words:**Cone-Beam Computed Tomography, Orthodontics, Impacted Tooth, Root resorption.

## Introduction

An impacted tooth might be defined as a tooth that has failed its eruptive movement, from its development location in the alveolar process into its proper location in dental arch within its normal period of growth and development, and that it won’t apparently full erupt based on clinical or radiographic assessment (1-3).

Impacted Maxillary Canines (IMC) is a relatively common pathology, having the third highest incidence ranging from 1% to 3%, whereas the incidence of impacted mandibular canine is one of the lowest (0,35%). IMC generally occupies a palatal position (85%) instead of a vestibular one (15%). These impactions are more commonly found in female patients (1,17%) than in male ones (0,51%) with a 2:1 ratio. Among all patients with IMC, only 8% have bilateral impactions. As for prevalence ratings, IMC ranges from 0.92% to 4.3% (2,4-9).

Permanent maxillary canine take a crucial role regarding masticatory function, dento-facial aesthetic and harmony and dental occlusion and stability (5). Therefore, it’s important that clinicians are aware of this condition in order to detect it as early as possible allowing a more correct diagnosis and treatment planning and preventing some of the possible complications associated with its occurrence and/or treatment (3,5,10).

Though it’s not defined a minimum of record set necessary for the orthodontic treatment(11), several radiographic projections might be needed in order to determine the exact position of an impacted tooth, exposing the patient to a high dose of radiation (1,4,5,11-14).

Discovered in the late 90’s by Professor Mozzo from Verona University, Italy, the Cone Beam Computed Tomography (CBCT)(15) allows patient’s study in three orthogonal planes (sagittal, coronal and axial), improving diagnosis and treatment planning not only in orthodontics but in several dentistry areas (4,16-20).

CBCT advantages over conventional 2D radiographs or CT exam are well known and its use in the study of IMC is not new. Yet the literature is not concise in what specific topics CBCT exam is strongly better than the conventional exams, justifying its use.

The aim of this study was to compare and conclude in what way the opinion regarding upper canine impaction location, adjacent tooth resorption, prognosis, image information, treatment plan and difficulty level could vary when observing a panoramic image compared to the observation of a set of CBCT reconstructions.

## Material and Methods

The study sample was based on the analysis of CBCT exams database of the Department of Dentistry, Faculty of Medicine, University of Coimbra (DDFMUC) where the selected patients had already been submitted to CBCT exam due to previous clinical indication for 3D evaluation.

Once applied the inclusion criteria – pre-existing CBCT from DDFMUC’s database; upper canine impaction (left or right, uni or bilateral); age over 13 years; 0,3mm voxel size of CBCT exam and FOV of 100mm – and exclusion criteria – syndromic patients or with craniofacial or dental anomalies that could affect tooth eruption and development; previous or current orthodontic treatment when CBCT scan was performed and artifacts that unable the CBCT analysis – 20 patients were included in the study, aged between 13 and 73 years old. A total number of 28 upper impacted canines were examined.

The selected patients were asked to sign an Informed Consent, in accordance with the Ethics Committee DDFMUC requirements. All patients’ privacy and confidentiality was kept in this study.

**Figure 1:**
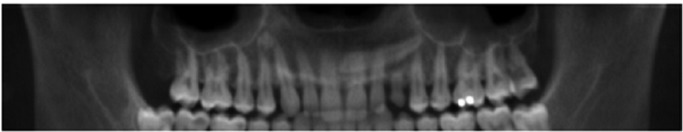
Example of panoramic reconstruction obtained from iCATVision, showing the ROI.

**Figure 2:**
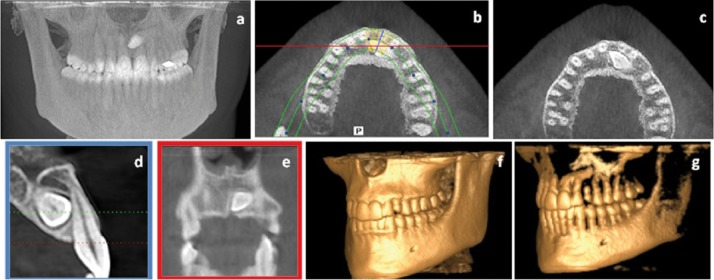
Example of images data set obtained from iCATVision software: (a) frontal cephalometric projection, (b) axial/transversal view showing the cuts used to reconstruct the sagittal view (represented by the blue line) and the coronal/frontal view (represented by the red line), (c) the same axial/transversal view without the lines, (d) sagittal view, (e) coronal view, (f) 3D reconstruction with a high level of bone density, and (g) the same 3D reconstruction with less bone density.

Patients were scanned with iCAT scanner machine (Imaging Sciences International, Hatfield PA, USA), set at 0,3mm voxel size, 120 kV tube voltage, 5mA current, 100 FOV, 4s of time scanning and with a slice thickness interval of 1,20mm. The CBCT images were then exported in format of Digital Imaging and Communications in Medicine (DICOM) and imported into iCATVision software (Imaging Sciences International, Hatfield PA, USA) for analysis. Several projections were reconstructed having two groups of images available for each impacted canine:

• Group A: a panoramic reconstruction image. This image was automatically reconstructed by the software based on anatomic landmarks and then cropped into the Region of Interest (ROI) (Fig. 1);

• Group B: a set of 7 reconstructions under different planes (Fig. 2).

One operator performed these segmentations for every case. Then each image was extracted from the software, saved as a JPEG file and printed in a high quality paper (Inapa Techno, Hamburg, Germany) using Ricoh MP C4500 (Ricoh Americas Corporation, Malvern PA, USA) laser printer. For every case, both gender and age was indicated.

A questionnaire was distributed to nine orthodontists, where they were asked to analyze ten different topics regarding the IMC ( Table 1). The exact same questions were applied to both groups A and B, after a random distribution of both groups’ images using an online sorter (https://www.random.org/) so that images from group A and B did not necessarily correspond.

**Table 1:**
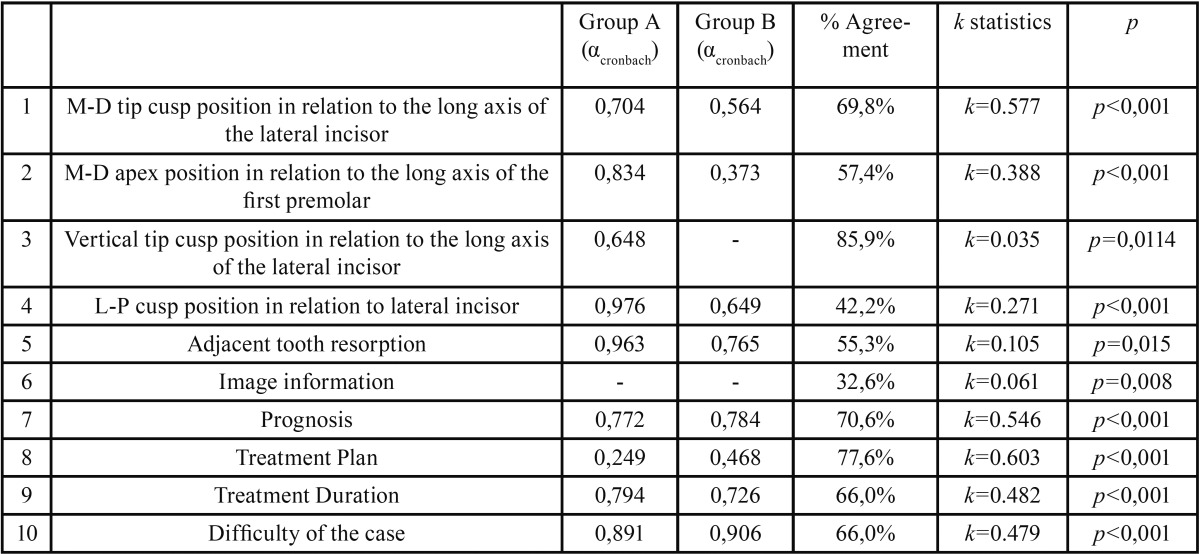
Summary of the results for every topic evaluated of the inter-rater reliability (αcronbach) for both groups A and B separately, the percentage of agreement between groups A and B, the kappa value and p value. The agreement percentage was obtained by the addition of the number of cases which maintained their classification.

The data set was analyzed using the SPSS software (version 22, SPSS Inc., USA). Statistic analysis was performed using Cronbach’s alpha statistics to analyze inter-rater reliability for each group (separately). Intra-rater agreement between the two exam modalities was measured using Kappa statistics for categorical questions and McNemar test for dichotomous questions, considering *p*<0,05 statistically significant.

## Results

The number of patients included in this study were 20, 50% (n=10) were males and 50% (n=10) were females, resulting in 1:1 ratio. A total of 28 IMC were found: 40% (n=8) were bilateral impactions, 30% (n=6) were unilateral right impactions and 30% (n=6) were unilateral left impactions. Patients included in the study were aged between 13,00 years and 73,08 years old, with a mean age of 26,83 years and a standard deviation of 16,43 years. The nine observers were all postgraduates in orthodontics.

Considering that there were nine observers and 28 canines to compare with both panoramic and CBCT images, the total number of data set was 252.

The results obtained are summarized in Table I where values obtained from the Cronbach’s Alpha test (αcronbach), the agreement percentage (% Agreement) found between the two methods, kappa values (k) and the significance level (p) are shown.

1) Canine localization in three orthogonal planes

Differences were found in the location of the IMC position between the two radiographic modalities. For the mesio-distal cusp position a strong 69,8% (n= 169) agreement between the two images was found between raters. With the analysis of CBCT images, the classification as “overllaped” was half the value (15,3%) found with the panoramic image (32,6%).

The mesio-distal apex position had an intra-rater agreement between panoramic and CBCT images of 57,4% (n=139). More than a third of the sample (n=98), were classified as “distal” with the panoramic image, whereas with CBCT images there was a significant change of answers to a more “mesial” or “overlapped” position”. Very few cases were classified as “NA” with both methods ( Table 2).

**Table 2:**
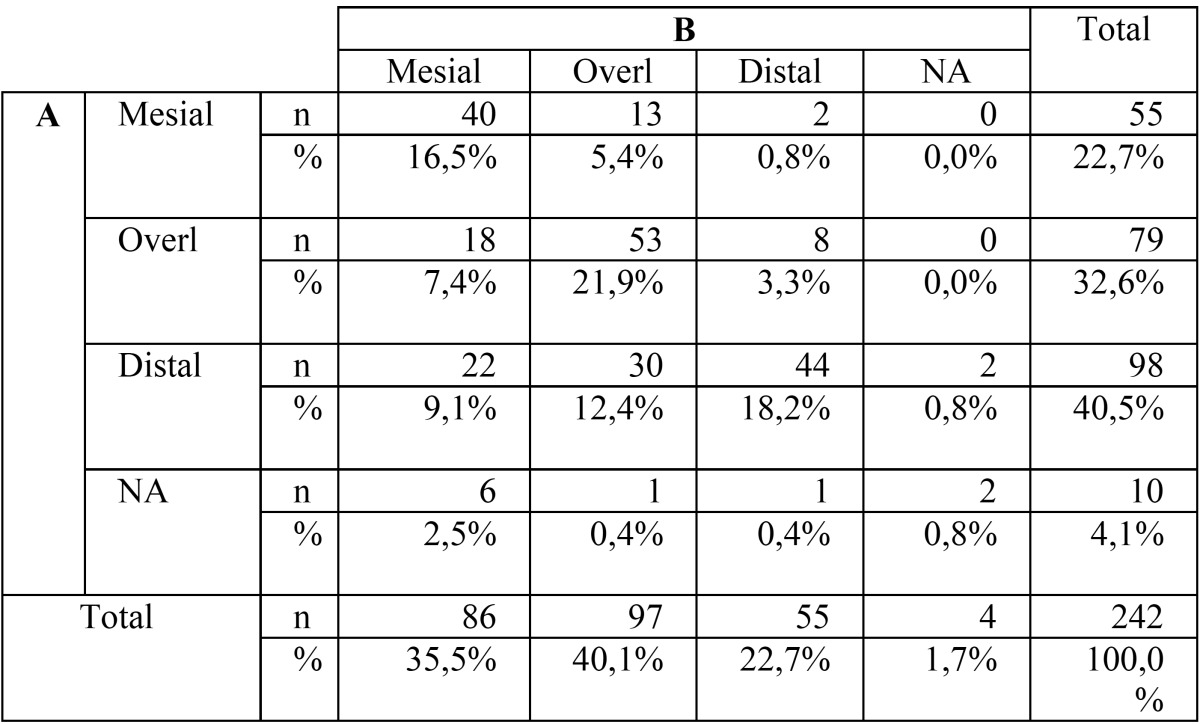
Crosstabulation for the M-D apex position.

Vertical cusp position had a high intra-rater agreement (85,9%), with almost every answers being classified as “bellow apex” and a very low number of cases classified as “NA” for both groups A and B.

Labio-Palatal (L-P) position had a significant poor intra-rater agreement (k=0.271; *p*<0,001) with an agreement percentage of only 42,2%. With the panoramic image in 36,8% of the cases (n=89) wasn’t possible to determine the IMC L-P position. This evaluation decreased significantly with the CBCT data set to only 17 cases. Also a higher “Labial” classification was found with the CBCT data set ( Table 3).

**Table 3:**
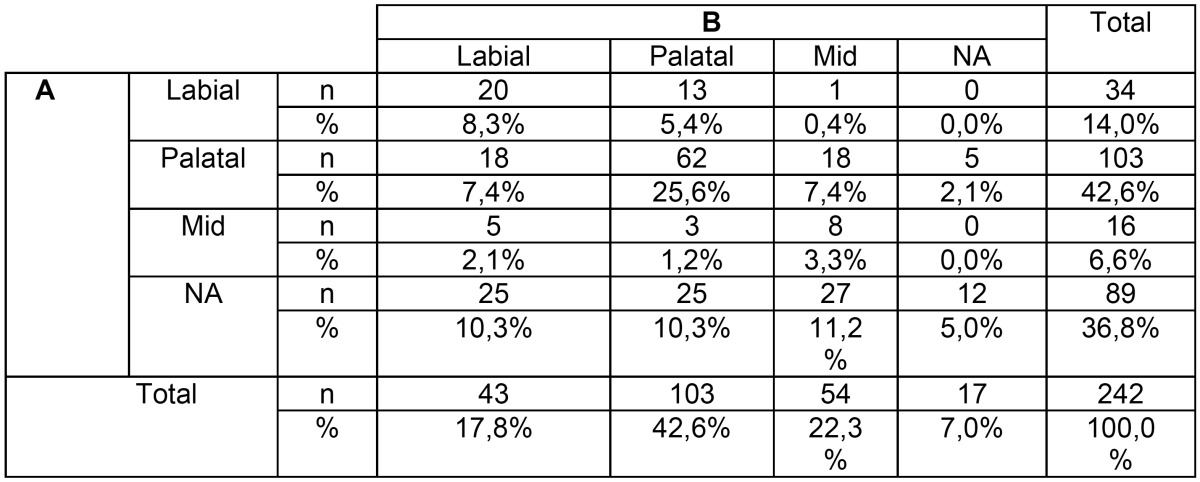
Crosstabulation for the L-P cusp position.

2) Adjacent tooth resorption

Observers were asked either if root resorption was present or not. The kappa statistics demonstrated a very poor agreement between the two groups (k=0.105; *p*=0,015), with CBCT analysis showing a lower classification of root resorption. The number of cases classified as “NA” decrease from 47 cases with the panoramic image to only 6 cases with CBCT analysis ( Table 4).

**Table 4:**
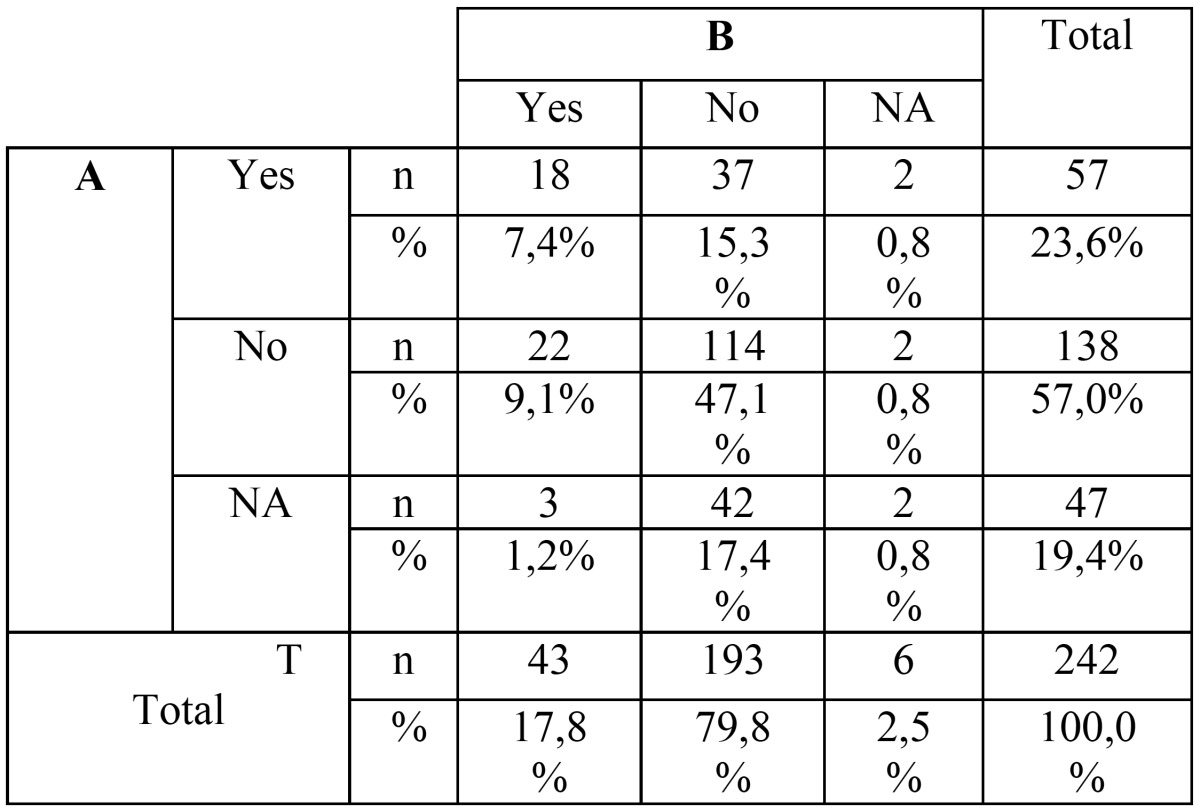
Crosstabulation for adjacent tooth resorption. Ten missing cases were observed and the percentage shown is considering as 100% the valid number of cases (n=242). NA: Non Available, n: number of cases, %: agreement percentage.

3) Image information

**Figure 3:**
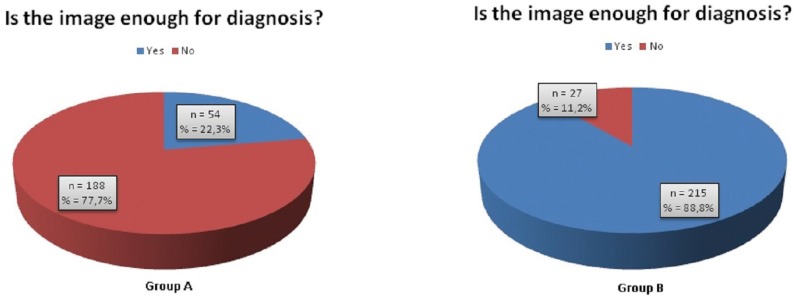
Pie chart indicating the answers obtained for the image information for group A (left) and group B (right).

The examiners were asked if the images were enough for orthodontic diagnosis. McNemar’s test shows that there is a statistically significant change of answers between groups (60,7% Cl95% [56,3%; 61,5%]; *p*=0,001). More than half of all cases scored as “No” with the panoramic image were changed when CBCT data set was available (Fig. 3).

4) Prognosis

When asked to classify cases prognosis a statistically significant moderate agreement was found between the two methods (k=0.546; *p*<0,001).

5) Treatment Plan

The orthodontists were given 4 treatment options: either to extract the deciduous canine, to perform orthodontic treatment with permanent canine traction, to extract permanent canine or not to treat. Inter-rater reliability was poor with the panoramic image (αcronbach) = 0,249 whereas with the CBCT views was moderate (αcronbach) = 0,468. The total percentages of answers were similar between the two groups. For both groups, the most frequent response was “orthodontic treatment with permanent canine traction”.

6) Orthodontic Treatment Duration

When asked about the orthodontic treatment duration, a strong agreement percentage was found between the two methods. However, in group B images, a slight longer treatment was scored than in group A.

7) Difficulty of the case

Lastly, the observers were asked to classify the case difficulty as: easy, moderate or difficult. A statistically significant moderate agreement was found between the two methods (k=0.479; *p*<0,001), with a 66,0% (n=159) percentage of agreement. A slightly lower score of “difficult” was obtained with the panoramic image compared with the CBCT data set (59 cases to 70 cases, respectively) but the majority of the answers were for both groups “moderate”.

## Discussion

The differences found in this study for the number of male and female patients (ratio 1:1) and the frequency of unilateral and bilateral impactions (60% and 40% respectively) from what’s described in literature (6,9), might be explained by the reduced sample size.

Regarding M-D cusp position, the higher classification as “overlapped” with panoramic data might be justified due to superimposition associated to this image. Haney *et al.* (4) found in their study a similar agreement percentage (79%) when comparing 2D radiograph and 3D CBCT volumetric views.

The results obtained for the M-D position of the apex were different than the ones described by Botticelli *et al.* (13). Though the author also found a significant lack of agreement between the two methods, our results show a higher tendency to score the apex tooth in a more distal area with the panoramic data. Also a very small number of cases were classified as “NA” for both panoramic and CBCT images in our study, whereas in the study of Wriedt *et al.* (5), in more than 25% of the cases, canine apex was not identified in the OPG. This can be justified with the reduction of the horizontal distortion provided by the panoramic CBCT reconstruction used in this study (21,22).

The agreement percentage found for the vertical tip cusp position (85,9%) was higher than the 50% of agreement described in prior a research by Haney *et al.* (4). In this question, for both panoramic image and CBCT reconstructions, there were almost zero cases classified as “NA”, suggesting that both exams allow the determination of the vertical cusp position.

Knowing the exact L-P position of the IMC is one of the most important questions either for the surgical exposure and to define the vector of traction (4,5,13). A statistically significant lack of agreement was found between the two sets of images, similar to a previous study (13) with only a 42,2 percentage of agreement, a very lower value than the 84% of agreement found in Haney *et al.* (4) study. A superior score for labial cusp location was verified with CBCT images. Also a significant decrease of “NA” classification was observed with CBCT data set compared to panoramic images, where, similarly to Wriedt *et al.* (5) study, more than third of the cases were scored as “NA” , suggesting that this data provides a better assessment of L-P cusp position.

The most common complication of canine impaction is resorption of the maxillary lateral incisor. In this study a decrease of the “NA” answers was verified with CBCT, compared to panoramic image. Tough the majority of the cases were assorted as having no root resorption, for both groups A and B, previous studies showed that 3D images are more sensitive and provide a better detection of root resorption than conventional 2D exams (5,23).

When the observers were asked about the image quality, a very high inter-rater reliability for both sets of images was found, just like a previous report (13). A great majority considered the panoramic image as insufficient for orthodontic diagnosis, whereas almost 90% of CBCT images were classified as sufficient for the same purpose.

In the analysis of treatment planning, a strong agreement was found between the two groups, similarly to Alqerban *et al.* (23) research, meaning that the treatment plan proposal didn’t differed much based on the panoramic and the CBCT data set. For both methods, orthodontic treatment with permanent canine traction was the preferred treatment plan. Some other studies found treatment plans to be different when analyzing 2D and 3D images (4,5,13).

The position of the impacted tooth and the inclination of its long axis strongly influence the prognosis, treatment duration and the difficulty of the case (3,24,25). In this study, the prognosis, treatment duration and difficulty of the case didn’t differ much between the two groups, what might be explained by the agreement found between the two groups for the treatment plan. However, a previous study (13) found that the difficulty of the case differed significantly comparing 2D and 3D images, with a higher degree of difficulty obtained with the 3D image set.

Summarily, the literature regarding IMC location with CBCT shows different results among all of these topics evaluated, what is very likely related with the lack of standardized methodologies. The results found in this study indicate that the greater differences between the two exams modalities are related with the mesio-distal apex position, the labio-palatal cusp position and with adjacent tooth resorption assessment, what might be explained with lack of 3D information of the panoramic image, suggesting CBCT examination when these issues are doubtful.

Further investigation, using precise protocols, should be done in order to evaluate in what cases CBCT exam has a clear advantage over conventional 2D exams, justifying its use.
